# Nutritional Quality, Bioactive Compounds, and Functional Characteristics of Guar ( *Cyamopsis tetragonoloba* L.) Genotypes: Multi‐Trait Selection of Superior Genotypes Using MGIDI Index

**DOI:** 10.1002/fsn3.72224

**Published:** 2026-08-06

**Authors:** Warqaa Muhammed ShariffAl‐Sheikh, Hussam F. Najeeb Alawadi, Ahmed Fahem Jabbar, Heidar Meftahizade

**Affiliations:** ^1^ Faculty of Basic Science Branch, Faculty of Dentistry University of Al‐Qadisiyah Al‐Qadisiyah Iraq; ^2^ College of Agriculture, University of Al‐Qadisiyah Al‐Qadisiyah Iraq; ^3^ Department of Horticultural Sciences, Faculty of Agriculture & Natural Resources Ardakan University Ardakan Iran

**Keywords:** dry matter digestibility, genetic diversity, gum viscosity, MGIDI, protein, seed gum

## Abstract

Guar ( *Cyamopsis tetragonoloba* L.) is a valuable and multifunctional summer legume crop that is widely exploited for seed gum extraction due to the presence of galactomannan in the seed endosperm, as well as for its protein‐rich by‐products (germ and hull), which are extensively used in various industries. The objective of this study was to conduct a comprehensive evaluation of the nutritional value, bioactive compounds, and functional characteristics of seeds from fifteen guar genotypes in order to identify superior genotypes for breeding programs and industrial applications. In this study, a wide range of key traits was assessed, including seed gum percentage (SG), gum viscosity, dry matter digestibility (DMD), protein, fiber, total oil, ash, tannin, total phenolic content (TPC), flavonoids, total antioxidant capacity (TAC), and carotenoids. Seed gum content varied from 25.7% in the Pishen genotype to 32.8% in the RGC‐986 genotype. Similarly, gum viscosity ranged from 2353.67 to 3466.67 cP, with the highest value observed in RGC‐986. Dry matter digestibility differed markedly among genotypes, ranging from 43.37% in Pishen to 51.97% in RGC‐986, reflecting genotypic variation in seed nutritional quality. Fiber and protein contents also showed considerable variability, with ranges of 10.57%–12.95% and 25.33%–33.33%, respectively, and RGC‐986 exhibited the highest values for both traits. Antioxidant‐related attributes, including TAC and carotenoids, varied significantly among genotypes; to enable simultaneous selection of superior genotypes based on multiple nutritional and functional traits, the multi‐trait genotype–ideotype distance index (MGIDI) was applied. According to this index, genotype BR‐99, with the lowest MGIDI value (2.04), was identified as the most desirable genotype, followed by RGC‐986 with a value of 2.45. Overall, the findings of this study highlight the considerable genetic variability among guar genotypes in terms of nutritional, biochemical, and functional traits, and demonstrate the potential of exploiting this diversity in breeding programs aimed at developing guar cultivars with enhanced nutritional value and improved industrial performance.

## Graphical abstract

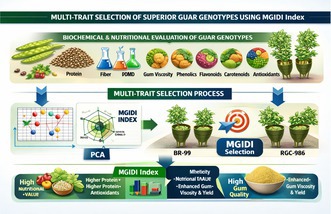

## 1 Introduction

Guar ( *Cyamopsis tetragonoloba* ) is an annual herbaceous species belonging to the Fabaceae family, traditionally cultivated for the production of guar gum, a natural polysaccharide with extensive applications in food and industrial sectors (Tahmouzi et al. 2023). Guar is well recognized for its relatively high tolerance to drought and its adaptability to arid and semi‐arid environments, making it particularly suitable for cultivation under hot and dry climatic conditions (Ashraf et al. 2002; Li et al. 2023). Guar is primarily cultivated for its seeds, which are rich in galactomannan (guar gum). This natural gum is widely used in the food industry as a thickening, stabilizing, and emulsifying agent in a variety of products, including sauces, ice cream, beverages, and animal feed (Malani et al. 2024). Moreover, young guar pods are consumed as a vegetable or used as livestock fodder in certain regions, highlighting the nutritional value of the plant beyond its gum production (Tahmouzi et al. 2023).

Guar possesses high nutritional and functional value due to the presence of galactomannan‐rich gum in its seeds. This natural polysaccharide is recognized as a source of soluble dietary fiber and has been shown to contribute to improved gastrointestinal function and glycemic regulation (Tahmouzi et al. 2023). Beyond its functional properties, guar gum is nutritionally important as a soluble dietary fiber, since soluble fibers are associated with reduced blood cholesterol levels, improved glycemic control, and enhanced satiety effects widely reported in studies addressing dietary fiber and metabolic health (Tahmouzi et al. 2023). Guar is recognized as a food resource with a complex composition of protein, dietary fiber, and bioactive compounds. Its seeds contain not only considerable amounts of protein and fiber but also diverse phenolic and flavonoid compounds that enhance its overall nutritional value (Gresta et al. 2018). Phenolic constituents such as gallic acid, chlorogenic acid, and other polyphenolic derivatives have been identified in guar seeds, and these compounds play a significant role in scavenging free radicals and exerting antioxidant effects, which may contribute to reducing the risk of chronic diseases associated with oxidative stress (Sharma, Joshi, et al. 2017; Sharma, Kaur, and Kaur 2017). The protein fraction of guar is notable not only for its relatively high concentration but also for the favorable amino acid profile, good digestibility, and functional properties of its protein isolates. These characteristics make guar protein a promising ingredient for the development of plant‐based functional and protein‐enriched food products (Kotnala et al. 2024).

The assessment and characterization of genetic diversity constitute a fundamental basis for crop improvement and development, as genetic variability serves as the primary source of heritable variation and beneficial traits, enabling the selection and breeding of superior cultivars (Manivannan et al. 2015; Vahdati et al. 2023; Habibi et al. 2023). The presence of genetic diversity within germplasm resources allows breeders to develop new varieties with desirable attributes such as resistance to pests and diseases, tolerance to environmental stresses, and improved yield performance, which are recognized as key challenges in sustainable agriculture (Manivannan et al. 2015; Farajpour 2025). In breeding programs, a precise understanding of genetic diversity among different genotypes, including guar, facilitates the identification and utilization of favorable genetic combinations tailored to specific nutritional, functional, or environmental objectives. This approach ultimately enhances the efficiency and success of developing high‐quality cultivars with improved agronomic and nutritional traits.

Given the economic importance and multifunctional applications of guar, investigating genetic diversity and genotype‐dependent variation in its biochemical and functional traits provides an effective approach for identifying genotypes with superior nutritional value and industrial potential. Traits such as seed gum content (SG) and viscosity play a decisive role in determining the industrial quality of guar gum, whereas protein, fiber, and carotenoids are considered key indicators of nutritional quality and health‐promoting properties. Recently, ShariffAl‐Sheikh et al. (2026) evaluated the same panel of guar genotypes under different irrigation regimes and identified drought‐resilient genotypes using physiological and biochemical traits combined with the MGIDI index. However, that study primarily focused on drought tolerance and stress physiology. In contrast, the present study provides the first comprehensive characterization of the nutritional quality, bioactive compounds, and functional properties of seeds under non‐stress conditions, including seed gum quality, total oil, ash, dry matter digestibility, and food‐related biochemical traits. Consequently, the superior genotype identified in this study differs from that selected for drought tolerance, highlighting distinct breeding objectives.

The primary objective of this study was to conduct a comprehensive evaluation of nutritional, biochemical, and functional traits in fifteen guar genotypes, with particular emphasis on economically and nutritionally important components, including seed gum content, viscosity, protein content, fiber, total oil, tannin, total phenolic content (TPC), flavonoids, total antioxidant capacity, carotenoids, ash content, and dry matter digestibility. By integrating precise quantitative and qualitative trait measurements with an assessment of genotypic variability, this research not only facilitates the identification of superior guar genotypes but also provides valuable applied insights for the development of improved cultivars with enhanced nutritional quality and industrial applicability. Ultimately, the findings of this study are expected to strengthen multi‐trait selection strategies in guar breeding programs and contribute to the advancement of food, pharmaceutical, and industrial products derived from guar.

## 2 Material and Methods

### 2.1 Experimental Design and Raw Materials

Fifteen guar genotypes from India, Pakistan, and Iran in a farm trial (RCBD, *R* = 3) in a research farm (32°14′ N, 54° 45′ E, 1074 m) of Ardakan university, Yazd, Iran during 2024. The seeds were sown with 5 cm in the soil and in a 40 × 20 cm row spacing, so that the distance between plants in the row was 20 cm and the distance between the rows is 40 cm. Each experimental plot of 4 × 2 m was considered to be a total of 8 m^2^ and the planting method was flat. The plants were watered using the drip irrigation method. Five plants were randomly selected from each plot for nutritional and biochemical assays were collected, while for yield estimation, the marginal plants were removed. The seed‐related traits (gum percentage, viscosity, etc.) have been assayed after the seeds ripen and mature.

### 2.2 Guar Gum and Chemical Composition

Gum extraction from guar seeds was performed following the method of Sabahelkheir et al. (2012). Initially, the seeds were soaked in water for 7 h. They were then manually rubbed to separate the different seed components, including the hull, endosperm, and embryo. For preparation of the gum extract, the method described by Ganatra et al. (2013) was employed. Physicochemical parameters, including ash content and protein concentration (calculated as %*N* × 6.25), were analyzed in accordance with standardized procedures recommended by the American Association of Cereal Chemists (AACC 2000). Lipid content and crude fiber were quantified using automated Soxtech (Model 2045, Foss) and Fibertech (Model 2023, Foss) systems, respectively.

### 2.3 Viscosity Measurement

The viscosity of guar gum was determined following the method of Sabahelkheir et al. (2012). The extracted gum powder was dissolved in water to allow full hydration. The solution was then centrifuged at 5000 rpm for 15 min to remove impurities. The supernatant was mixed with ethanol in a 3:1 ratio and left for 50 min to weaken the interactions between water and gum molecules, allowing the gum to separate from the water. White fibrous strands of gum were observed, which were collected and dried in an oven at 40°C for 6 h to obtain purified guar gum. Solutions of varying concentrations (0.1%, 0.25%, and 0.5% w/w) were then prepared from the purified gum. For example, for the 0.5% solution, 0.5 g of guar gum powder was dissolved in 99.5 g of deionized water and stirred magnetically overnight to ensure complete hydration. The viscosity of the resulting solutions was measured using a Brookfield viscometer (Model DV3 Ultra, USA) following the procedure described by Casas et al. (2000).

### 2.4 Dry Matter Digestibility (DMD)

Dry matter digestibility was determined using the two‐stage in vitro method described by Tilley and Terry (1963). Samples were oven‐dried at 105°C to constant weight, ground, and passed through a 1 mm sieve. A 0.5 g portion of the dried sample was incubated under anaerobic conditions with buffer solution and fresh rumen fluid at 39°C for 48 h. The subsequent gastric digestion phase was carried out by adding a hydrochloric acid solution containing pepsin, followed by a second 48‐h incubation at the same temperature. At the end of the incubation, the residues were dried and weighed. Dry matter digestibility was calculated using the formula:

$$\begin{array}{cc} \mathrm{DMD}\;\left(\%\right) & =\left[\text{Initial}\;\mathrm{dry}\;\text{weight}-\text{Residual}\;\mathrm{dry}\;\text{weight}\right]/\\ & \left[\text{Initial}\;\mathrm{dry}\;\text{weight}\right]\times 100 \end{array}$$

### 2.5 Determination of Tannin Content

Initially, 0.2 g of the powdered seed sample was accurately weighed and extracted with 70% (v/v) acetone–water solution at a ratio of 3:1. The resulting mixture was centrifuged at 2000 rpm for 5 min to separate the precipitated materials. The residue was re‐extracted with 70% acetone. Subsequently, the combined extracts were partitioned into two phases by the addition of sodium chloride solution. The aqueous phase was further extracted with 70% acetone, after which the acetone was removed by evaporation. Then, 2 mL of distilled water was added to the remaining solution, and the extraction procedure was repeated three times. Residual solvent was removed from the liquid phase using a rotary evaporator under reduced pressure. Finally, the absorbance of the extract was measured at a wavelength of 765 nm using a spectrophotometer, following the method described by Price et al. (1979).

### 2.6 Total Phenolic Compound

Guar flour (500 mg) was extracted with 20 mL of methanol (with a distilled water ratio of 8:2 v/v) using 10 min of room temperature ultrasonic. The extraction process continued for another 20 h in darkness and at room temperature. The methanol extracts were centrifuged at 4000 rpm for 15 min. Folin Ciocalteu (Merck, Germany) was used as a reactive agent. An extract aliquot (100 μL) was applied to and blended with 2 mL Folin Ciocalteu reagent (1:10 diluted with distilled water) and 2.5 mL Na_2_CO_3_ aqueous 7.5 (w/v). The total phenolic content of the resulting solutions was calorimetrically assessed at room temperature at a wavelength of 765 nm (UV 2600; Shimadzu Corporation, Kyoto, Japan) after 80 min of incubation, in which gallic acid was added as normal (Singleton et al. 1999). Total phenolic content was measured as %.

### 2.7 Flavonoid Content

To evaluate the flavonoid amount, 1.5 mL of 95% ethanol, 0.1 mL of 10% aluminum chloride (AlCl_3_, 6H_2_O), 0.1 mL of sodium acetate (NaC_2_H_3_O_2_·3H_2_O) (1 mol/L), and 2.80 mL of distilled water were mixed with each 500 μL of the sample. Using a spectrophotometer, the optical intensity was measured by absorption at a wavelength of 415 nm. In a blank analysis, the same technique was used (Chang et al. 2002). The findings were represented in milligrams of equivalent quercetine per gram of dry material. The tests were done in triplicate.

### 2.8 Carotenoids

The total carotenoid content was determined according to the method of Lichtenthaler (1987) with slight modifications. 0.2 g of the dry powder of the sample was extracted in 10 mL of 80% acetone, and the resulting mixture was stirred for 15 min in the dark. After centrifugation at 3000 rpm for 10 min, the supernatant was collected. The absorbance of the extract at wavelengths of 470, 645, and 663 nm was measured using a spectrophotometer, and the total carotenoid content was calculated based on the standard Lichtenthaler equation. The results were reported as mg carotenoid per g fresh weight of the sample.

### 2.9 Total Antioxidant Capacity (TAC)

Using the 1,1‐diphenyl‐2‐picrylhydrazyl radical scavenging method, based on the hydrogenation ability of the extract: According to the method of Koleva et al. (2002), the DPPH level was measured. First, different concentrations of guar seed powder extracts were prepared, then 0.025 g/L of 1,1‐diphenyl‐2‐picrylhydrazyl was added to each of them. Then, they were kept at room temperature and in the dark for 30 min. A blank sample was also prepared. Its DPPH level was measured using a spectrophotometer at a wavelength of 517 nm. Quercetin solution was used as a standard sample. The percentage of free radical inhibition (% I) of each extract was calculated using the equation:

$$\mathrm{TAC}\;\left(\%\right)=\left(A\;\text{control}-A\;\text{sample}\right)/A\;\text{control}\times 100$$

### 2.10 Statistical Analysis

This experiment was conducted to evaluate 15 different guar genotypes. Analysis of variance (ANOVA) was performed in a randomized complete block design (RCBD) with three replications, and comparisons of means were made using Duncan's least significant difference test at a significance level of 5%. Statistical analyses were performed using SAS software. Multivariate analyses, including hierarchical cluster analysis (HCA) and heat map visualization, were performed using the factoextra and pheatmap packages in R. Pearson correlation coefficients were calculated to evaluate relationships between traits using the ggplot2 package for visual display. Principal component analysis (PCA) was performed to extract key traits and explain variance. In addition, the multi‐trait genotype–ideotype distance index (MGIDI) was used to systematically evaluate and rank genotypes based on multiple traits. An ideotype genotype was defined based on the desired values for each trait, and the distance of each. The genotype was calculated from this ideotype to produce the MGIDI index. The genotypes with the smallest distance showed the closest match to the ideotype. The results were used to rank the genotypes, identify the superior genotypes, and visualize using MGIDI index graphs (Olivoto and Nardino 2021).

## 3 Results

The MGIDI index was calculated following the methodology proposed by Olivoto and Nardino (2021). Prior to analysis, all traits were standardized to remove scale effects. Traits were defined based on breeding objectives, where seed gum, viscosity, protein, fiber, and antioxidant‐related traits were set to be maximized, while tannin content was minimized. A selection intensity of approximately 15% was applied, resulting in the selection of the top two genotypes. The ideotype was defined as a hypothetical genotype with optimal values for all desirable traits. No additional weighting was applied, and all traits contributed equally to the MGIDI index.

### 3.1 Descriptive Statistics

Evaluation of seed gum percentage (SG) among the studied genotypes revealed a remarkably wide range of variation, with values extending from 24.5 to 33.4 (Table 1). The mean SG was calculated as 28.17, while the relatively high standard deviation of 2.12 indicates substantial dispersion of this trait across genotypes. The coefficient of variation of 7.55% reflects pronounced genetic variability and highlights the considerable potential for selecting superior genotypes with enhanced seed gum content. With regard to dry matter digestibility (DMD), values ranged from 42.38 to 52.4. The mean DMD was 46.96, and the coefficient of variation of 5.9% reflects high uniformity and stability of this important nutritional parameter.

**TABLE 1:** Descriptive statistics of all traits in 15 genotypes of *Cyamopsis tetragonoloba* L.

Traits	Abb	Min	Median	Max	CV (%)	Mean	Std. deviation	Std. error
Seed gum (%)	SG	24.5	27.6	33.4	7.55	28.17	2.128	0.317
Dry matter digestibility (%)	DMD	42.38	46.5	52.4	5.9	46.96	2.773	0.413
Viscosity (1% cps)	Viscosity	2200	2530	3500	13.57	2645	359	53.520
Ash (%)	Ash	3.5	3.98	4.89	6.96	4.033	0.281	0.042
Total oil (%)	TO	1.8	2.6	3.5	17.13	2.591	0.4446	0.066
Fiber (%)	Fiber	10.2	11.87	13.2	5.51	11.71	0.6457	0.096
Tannin (%)	Tannin	1.9	2.45	2.98	8.78	2.434	0.2135	0.032
Protein (%)	Protein	24	28	35	9.19	28.07	2.58	0.385
Total phenolic phenol (%)	TPC	0.57	0.93	2.1	31.7	1.038	0.3327	0.050
Flavonoids (% dry matter)	Flavonoids	0.21	0.24	0.29	7.75	0.2458	0.01913	0.003
Carotenoids (mg/g FW)	Carotenoids	1.3	2.2	2.8	15.56	2.122	0.3323	0.050
Total antioxidant capacity (%)	TAC	41.5	45.2	48.2	3.81	44.9	1.604	0.239

*Note:* Baseline values presented in this table correspond to the non‐stress (100% field capacity) treatment previously reported by ShariffAl‐Sheikh et al. (2026) and are reproduced here to provide the descriptive statistics for the nutritional and functional analyses conducted in the present study.

Regarding viscosity (Viscosity), the recorded values ranged from 2200 to 3500. The mean viscosity was 2645, with a standard deviation of 359, and a coefficient of variation of 13.57%, revealing considerable differences among genotypes in terms of rheological properties. The results for ash content (Ash) showed a relatively narrow range from 3.5 to 4.89. The mean Ash was 4.033, and the low coefficient of variation of 6.96% indicates high uniformity and stability of mineral composition among the genotypes. In terms of total oil content (TO), minimum and maximum values of 1.8 and 3.5 were recorded, respectively. The mean TO was 2.591, accompanied by a coefficient of variation of 17.1%, reflecting moderate variability that may be relevant for improving nutritional quality. The evaluation of fiber content (Fiber) revealed values ranging from 10.2 to 13.2, with a mean of 11.71. The low coefficient of variation of 5.5% suggests high stability of Fiber and a limited contribution of genetic variability to this trait. For tannin content (Tannin), the observed values ranged from 1.9 to 2.98. The mean Tannin was 2.434, and the coefficient of variation of 8.78% indicates low to moderate variability among the genotypes. Analysis of protein content (Protein) indicated that this trait varied between 24 and 35. The mean Protein was calculated as 28.07, with a coefficient of variation of 9.19%, suggesting acceptable genetic diversity and favorable nutritional potential (Table 1). Assessment of total phenolic content (TPC) showed that this trait varied from 0.57 to 2.1 across the genotypes. The mean TPC was 1.038, with a standard deviation of 0.3327, reflecting appreciable variability in phenolic accumulation. A coefficient of variation of 31.7% further emphasizes the substantial diversity of TPC within the studied population. With respect to flavonoid content (Flavonoids), a narrow range of variation was observed, as values varied from 0.21 to 0.29. The mean flavonoid content was 0.245, accompanied by a low coefficient of variation of 7.79%, suggesting a relatively uniform distribution of this trait among the evaluated genotypes. Finally, examination of carotenoid content (Carotenoids) revealed extremely high variability, with values ranging from 1.3 to 2.8. The mean Carotenoids was 2.12, and the coefficient of variation of 15.6% indicates pronounced differences among genotypes, highlighting substantial breeding potential for enhancing the bioactive and antioxidant value of guar. Analysis of total antioxidant capacity (TAC) demonstrated noticeable variation, with values ranging from 41.5 to 48.2. The mean TAC was estimated at 44.9, and the coefficient of variation reached 3.81%, indicating moderate diversity in antioxidant potential among the genotypes (Table 1).

#### 3.1.1 Seed Gum Content

**FIGURE 1:**
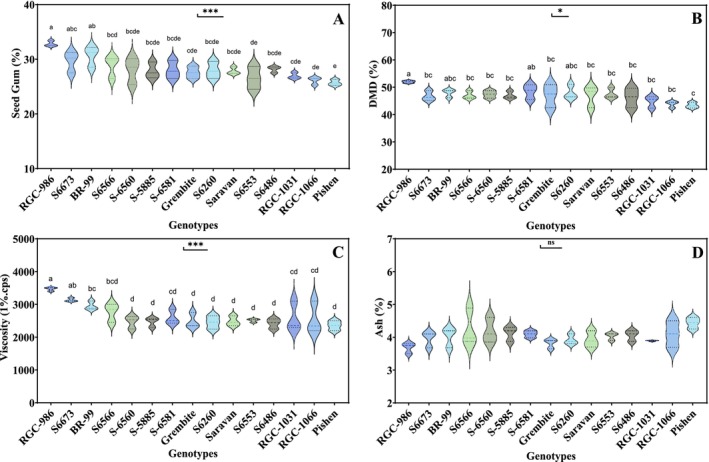
Evaluation of (A) seed gum, (B) dry matter digestibility (DMD), (C) viscosity, and (D) ash in 15 different guar genotypes. *p ≤ 0.05, **p ≤ 0.01, and ***p ≤ 0.001 and non‐significant (ns).

The evaluation of seed gum content (SG) across 15 guar genotypes revealed that values ranged from a minimum of 25.7 in Pishen to a maximum of 32.8 in RGC‐986 (Figure 1A). Other genotypes exhibited moderate to high levels; for instance, BR‐99 and S6673 recorded 30.6 and 29.63, respectively, placing them in the upper range, while S‐5885, S6566, and S‐6560 showed values of 27.87, 28.8, and 28. Genotypes S6581, S6486, and S6260 were positioned in the mid‐range with values around 28 to 27.96, and RGC‐1031, RGC‐1066, and Pishen displayed the lowest SG levels, at 26.93, 26.1, and 25.7, respectively.

#### 3.1.2 Viscosity

The assessment of viscosity (Viscosity) of seed gum among the 15 guar genotypes indicated a range from 2353.67 in Pishen to 3466.67 in RGC‐986 (Figure 1B). Genotypes S6673 and BR‐99 also exhibited high values of 3133.33 and 2950, ranking among the top performers, whereas S6566 and S‐6581 recorded moderate viscosities of 2766.67 and 2593.33. Genotypes S‐6560, S‐5885, Grembite, Saravan, S6260, S6553, S6486, RGC‐1031, and RGC‐1066 showed values between 2416.67 and 2583.33, representing mid‐ to low‐range levels. Pishen exhibited the lowest viscosity at 2353.67.

#### 3.1.3 Dry Matter Digestibility and Ash

The evaluation of dry matter digestibility (DMD) among guar genotypes showed values ranging from 43.37 in Pishen to 51.97 in RGC‐986. Genotypes BR‐99 and S6260, with 47.91 and 47.99, were above the average, while S6566, S‐6560, S‐5885, and S6553 recorded moderate digestibility values between 47.08 and 47.61. Genotypes S6581 and S6673, with 48.43 and 46.76, were positioned in the mid‐range, and Saravan and Grembite showed values of 46.92 and 46.97. Conversely, RGC‐1031 and RGC‐1066, with 44.83 and 43.81, along with Pishen at 43.37, exhibited the lowest DMD values (Figure 1C). The study of ash content across the 15 guar genotypes revealed values ranging from 3.68 in RGC‐986 to 4.43 in Pishen. Genotypes S6673, BR‐99, and S6566 showed higher values of 3.96, 4.03, and 4.25, respectively. Additionally, S‐6560, S‐5885, and S‐6581 recorded moderate to high levels of 4.18, 4.12, and 4.09. Genotypes Grembite, S6260, Saravan, and RGC‐1031 displayed mid‐range values between 3.82 and 3.89, while S6553 and S6486, with 4.03 and 4.06, were close to the overall mean. RGC‐1066, with a value of 4.10, was also positioned in the upper range (Figure 1D).

#### 3.1.4 Total Oil

**FIGURE 2:**
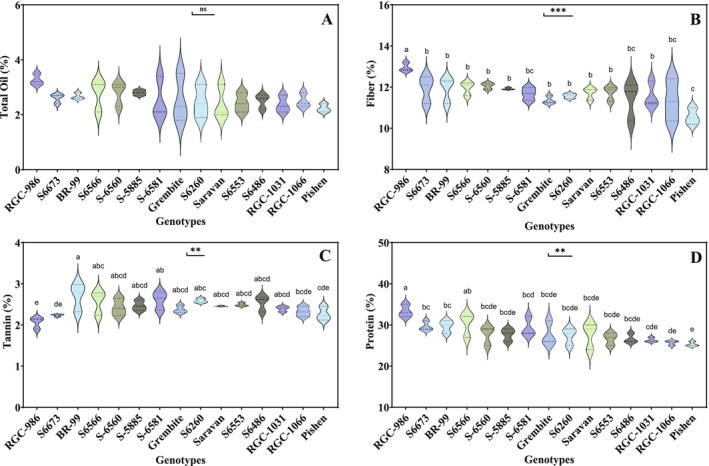
Evaluation of (A) total oil, (B) fiber, (C) tannin, and (D) protein in 15 guar genotypes. *p ≤ 0.05, **p ≤ 0.01, and ***p ≤ 0.001 and non‐significant (ns).

The analysis of total oil content (TO) among the 15 guar genotypes revealed values ranging from a minimum of 2.23 in Pishen to a maximum of 3.27 in RGC‐986 (Figure 2A). Genotypes S‐6560 and S‐5885, both with values of 2.80, were above the average, while S6566 and BR‐99 recorded 2.77 and 2.67, respectively, indicating satisfactory performance in total oil content. Genotypes S6673, S‐6581, Grembite, and S6486 exhibited mid‐range values between 2.50 and 2.60, whereas S6260, Saravan, and S6553 recorded lower values ranging from 2.43 to 2.47. RGC‐1031 and RGC‐1066, with 2.37 and 2.50, as well as Pishen with 2.23, displayed the lowest TO levels.

#### 3.1.5 Fiber

The evaluation of fiber content in the 15 guar genotypes indicated that values ranged from 10.57 in Pishen to 12.95 in RGC‐986. Genotypes S‐6560 and S6566, with 12.06 and 11.96, were above the mean, while S6673, BR‐99, and S‐5885 recorded values between 11.92 and 11.93, demonstrating notable fiber content. Genotypes S6553, Saravan, and S‐6581, with values of 11.73, 11.70, and 11.67, were positioned in the mid‐range, whereas S6260, RGC‐1031, S6486, Grembite, and RGC‐1066, with values between 11.35 and 11.58, exhibited moderate fiber levels. Pishen displayed the lowest fiber content at 10.57 (Figure 2B).

#### 3.1.6 Tannin

The analysis of tannin content among the 15 guar genotypes showed values ranging from 2.06 in RGC‐986 to 2.70 in BR‐99. Genotypes S6566 and S‐6581, with values of 2.57 and 2.60, were positioned in the upper range, while S‐6560, S‐5885, and S6553, with values between 2.43 and 2.49, exhibited moderate levels. Genotypes S6673 and Pishen, with 2.23 and 2.27, were in the lower range, whereas Grembite, RGC‐1031, RGC‐1066, and Saravan, with values from 2.33 to 2.46, showed moderate tannin content. S6260, with 2.59, was placed in the mid‐to‐high range (Figure 2C).

#### 3.1.7 Protein

The evaluation of protein content in the 15 guar genotypes revealed values ranging from a minimum of 25.33 in Pishen to a maximum of 33.33 in RGC‐986. Genotypes S6566 and BR‐99, with values of 30.33 and 29.67, were above the mean, and S6673, with 29.67, also showed favorable protein content. Genotypes S‐6581 and S‐6560, with 29.33 and 27.67, were positioned in the mid‐range, whereas Grembite, S6260, Saravan, and S6553, with values between 27.33 and 26.67, exhibited moderate to low protein levels. S6486, RGC‐1031, and RGC‐1066, with 26.33 to 25.67, as well as Pishen with 25.33, showed the lowest protein content (Figure 2D).

#### 3.1.8 Total Phenolic Content

**FIGURE 3:**
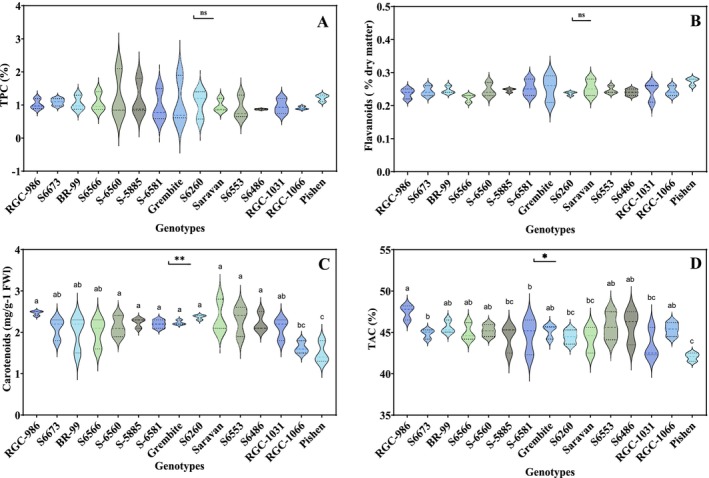
Evaluation of (A) total phenolic compounds (TPC), (B) flavonoids, (C) carotenoids, and (D) total antioxidant capacity (TAC) in 15 different guar genotypes. *p ≤ 0.05, **p ≤ 0.01, and ***p ≤ 0.001 and non‐significant (ns).

The analysis of total phenolic content (TPC) among the 15 guar genotypes revealed values ranging from a minimum of 0.87 in S6486 to a maximum of 1.26 in S‐6560. Genotypes Pishen and S‐5885, with values of 1.22 and 1.17, were also in the upper range. S6673, BR‐99, S6566, RGC‐986, and Grembite recorded values between 1.05 and 1.09, indicating moderate to high performance in TPC. In the mid‐range, S6260 and RGC‐1031 exhibited values of 1.06 and 0.96, while Saravan and S‐6581 showed 0.97 and 0.96. Genotypes S6553, RGC‐1066, and S6486, with values of 0.90, 0.91, and 0.87, were in the lower range for TPC (Figure 3A).

#### 3.1.9 Flavonoid Content

The evaluation of flavonoid content showed values ranging from 0.22 in S6566 to 0.27 in Pishen. Genotypes BR‐99, S‐6560, S‐5885, S‐6581, Grembite, and Saravan, all with values around 0.25, were above the mean, whereas RGC‐986, S6673, S6260, S6486, RGC‐1031, and RGC‐1066, with values of 0.24, showed relatively uniform flavonoid content. S6566 had the lowest flavonoid level at 0.22, while Pishen recorded the highest at 0.27 (Figure 3B).

#### 3.1.10 Carotenoids

The analysis of carotenoids revealed values ranging from 1.50 in Pishen to 2.47 in RGC‐986. Genotypes S‐5885, Grembite, and S6486, with values of 2.23, and S6553, Saravan, and S6260, with 2.30 to 2.37, were above the mean. S6673, S‐6560, and RGC‐1031, with values between 2.10 and 2.13, showed moderate performance, while BR‐99, S6566, and S‐6581, with values from 2.00 to 2.20, were in the mid‐range. RGC‐1066 and Pishen, with values of 1.63 and 1.50, exhibited the lowest carotenoid content (Figure 3C).

#### 3.1.11 Total Antioxidant Capacity

The assessment of total antioxidant capacity (TAC) indicated values ranging from 42.03 in Pishen to 47.50 in RGC‐986 (Figure 3D). Genotypes BR‐99, S6566, and S‐6560, with values of 45.61, 45.00, and 45.22, were above the mean, while S6553 and S6486, with 45.73 and 45.77, displayed the highest moderate values. S6673, S‐5885, S‐6581, Grembite, S6260, and Saravan, with values between 44.23 and 44.84, fell in the mid‐range. In contrast, RGC‐1031 and RGC‐1066, with 43.47 and 45.37, showed moderate to low TAC, whereas Pishen exhibited the lowest antioxidant capacity at 42.03.

### 3.2 Results of Principal Component Analysis

**FIGURE 4:**
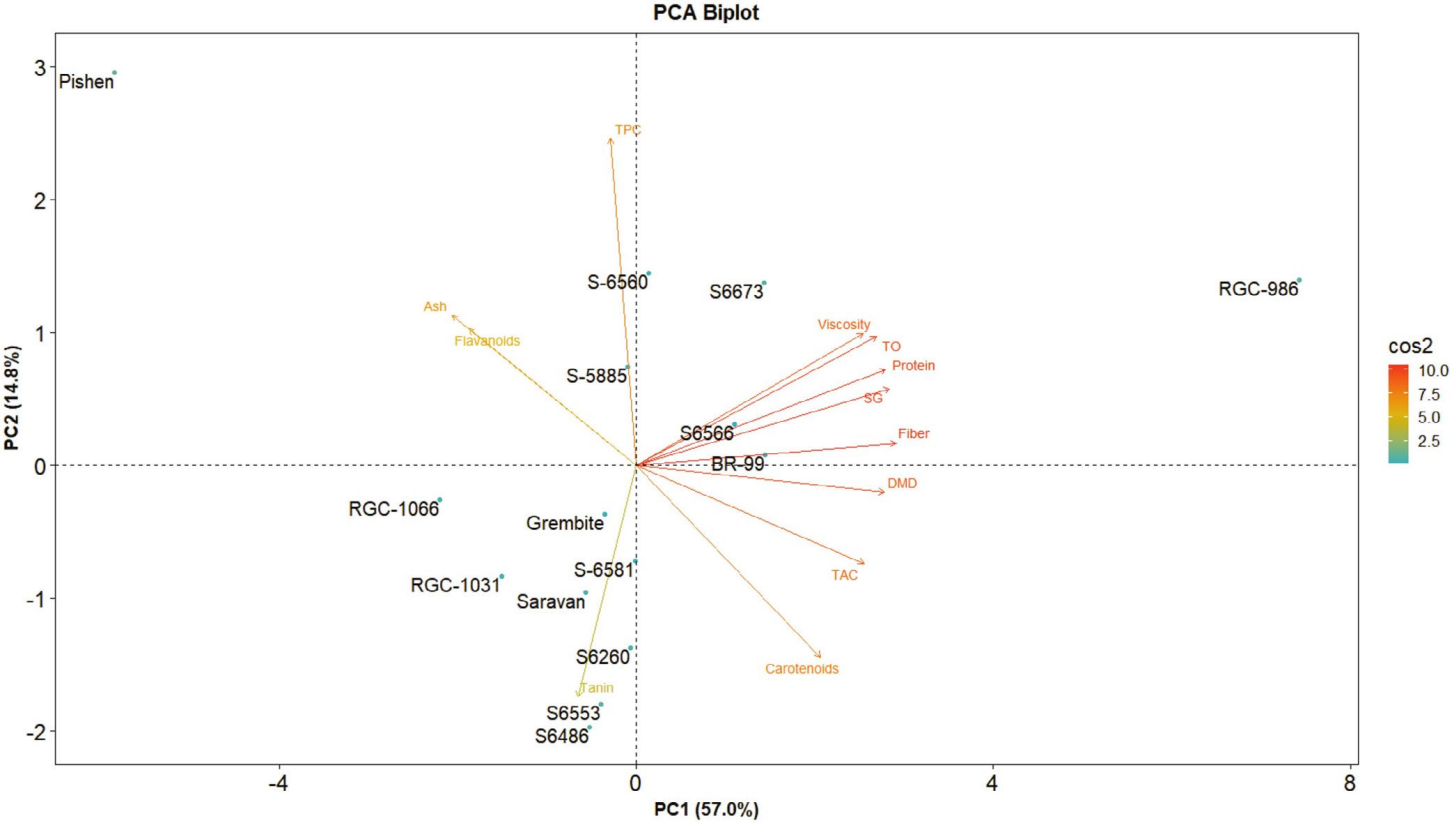
Principal component analysis (PCA) biplot illustrating the biochemical traits of 15 different genotypes of guar. The biplot displays the first two principal components (PC1 and PC2), which explain the major proportion of total variance among the measured traits. Circles represent the sample scores, while arrows indicate the loading vectors corresponding to each trait. For the definitions of the measured traits and treatments, refer to Table 1.

The principal component analysis (PCA) conducted in the present study indicated that the first two principal components, PC1 and PC2, explained 57% and 14.8% of the total variance, respectively, demonstrating their strong capacity to discriminate among the measured variables and genotypes (Figure 4). Tannin showed negative loadings on both PC1 and PC2. Total phenolics and flavonoids exhibited positive loadings on PC2, while their contributions to PC1 were close to zero. Variables such as carotenoids, total antioxidant capacity (TAC), and dry matter digestibility (DMD) were positioned in the lower positive region of the biplot. In contrast, traits including viscosity, total oil (TO), protein, seed gum (SG), and fiber were mainly distributed in the positive quadrant of PC1. Regarding the dispersion of the studied genotypes, RGC‐986 and Pishen were clearly separated from the remaining genotypes and appeared at the greatest distance from both the cluster center and other genotypes, indicating their distinct biochemical and nutritional profiles.

### 3.3 Heatmap Cluster Analysis

**FIGURE 5:**
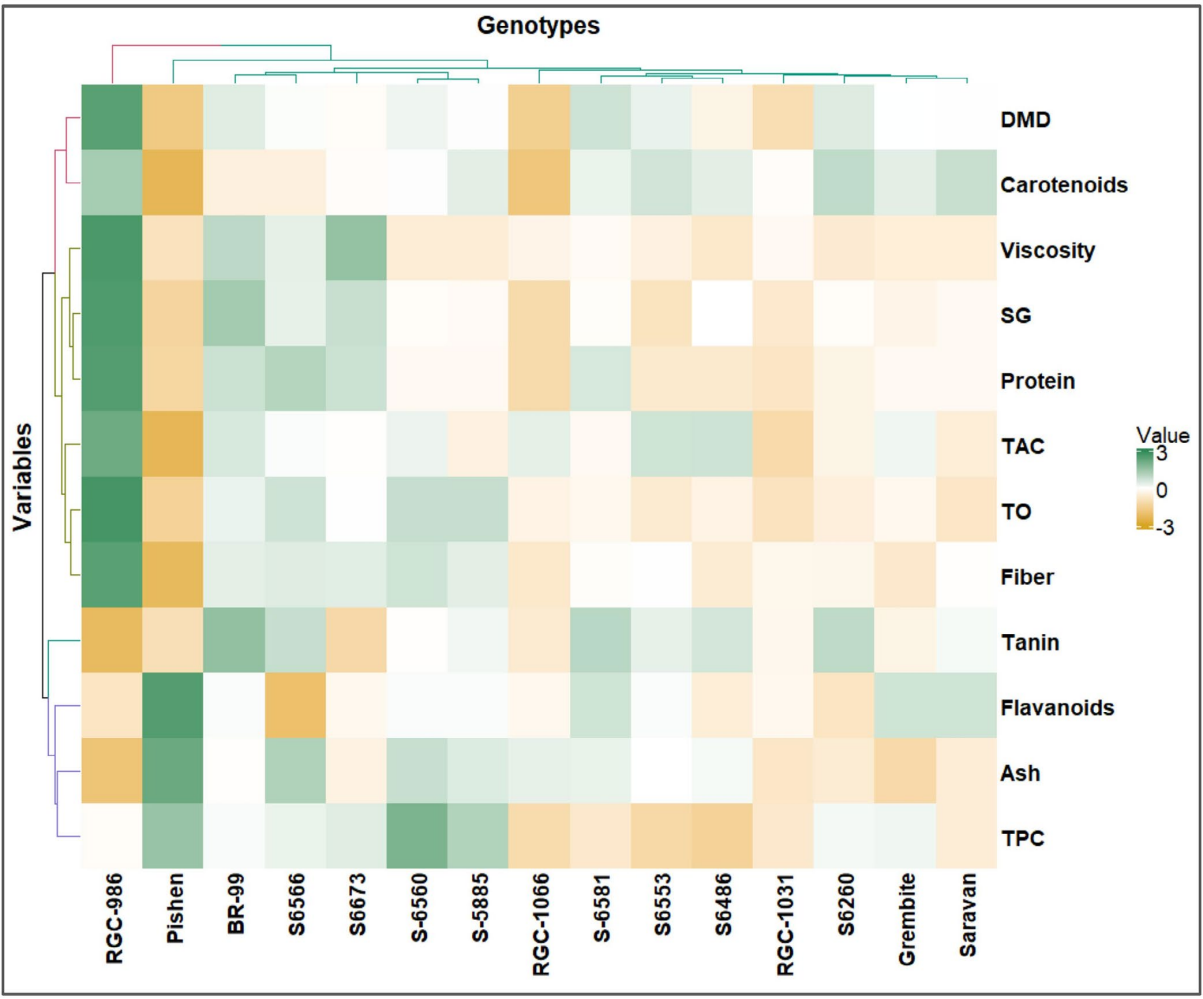
Heatmap cluster analysis of biochemical and functional traits in 15 guar genotypes.

In the present study, clustering of the evaluated traits and genotypes was performed using cluster analysis based on a heatmap (Figure 5). The results indicated that the studied traits were grouped into two main clusters, each further divided into several sub‐clusters. Traits such as total phenolic content (TPC), flavonoids, ash, and tannin were grouped within one main cluster, with tannin forming a distinct and separate sub‐cluster on its own. In addition, the analysis showed that fiber, total oil (TO), and total antioxidant capacity (TAC) were grouped into one sub‐cluster, while protein, seed gum (SG), and viscosity were clustered together in another sub‐cluster. Carotenoids and dry matter digestibility (DMD) also formed a separate sub‐cluster. Regarding genotype classification, the results demonstrated that genotype RGC‐986 was placed in an independent cluster, whereas the remaining genotypes were grouped together into different clusters and sub‐clusters, reflecting varying degrees of similarity among them.

### 3.4 Multi‐Trait Genotype–Ideotype Distance Index

**FIGURE 6:**
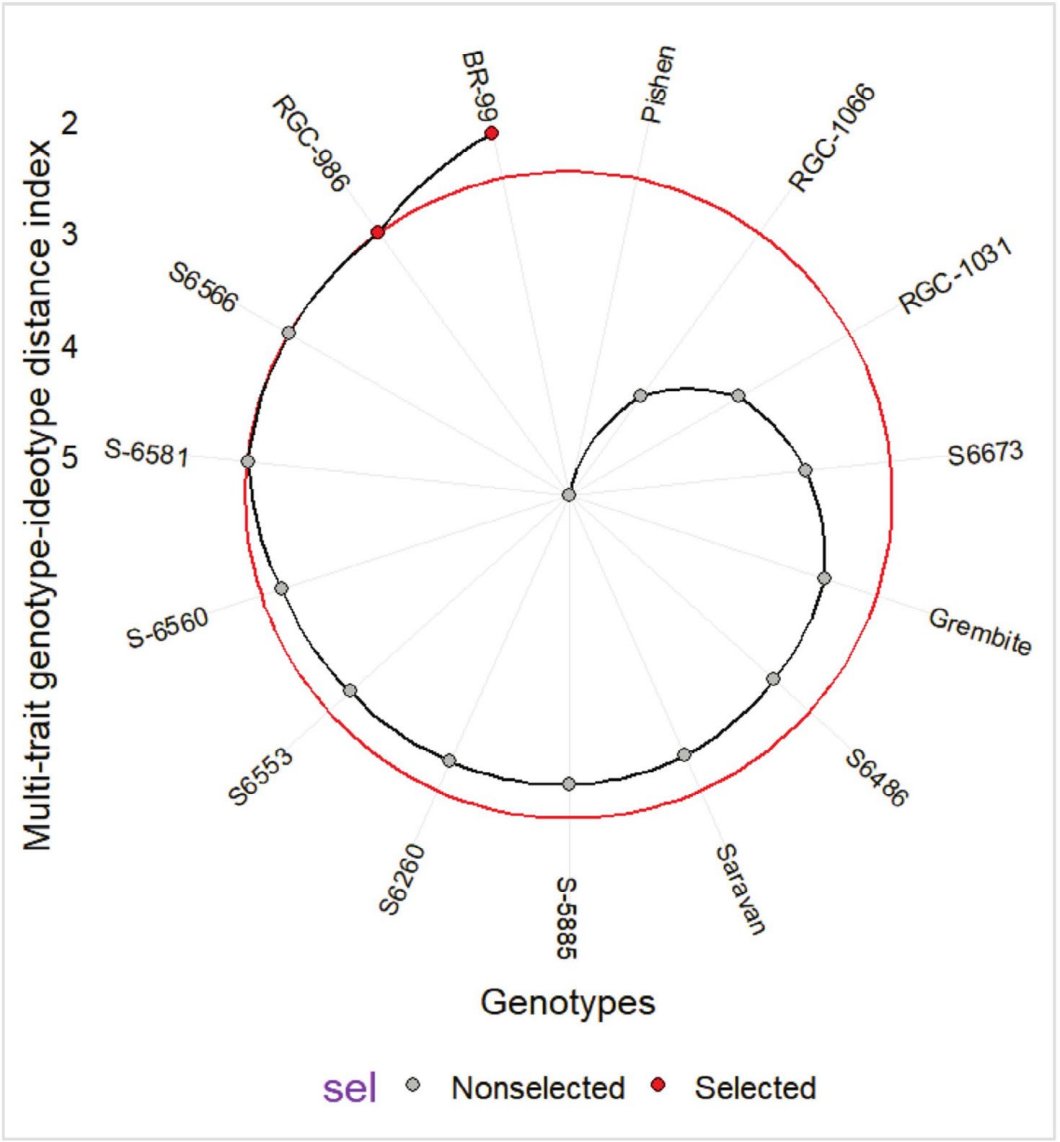
Genotypes ranking of 15 genotypes guar ( *Cyamopsis tetragonoloba* L.) based on the MGIDI index, with selected superior genotypes highlighted in red.

The results of the Multi‐trait Genotype–Ideotype Distance Index (MGIDI) for ranking guar genotypes indicated a relatively wide range of variation among the evaluated genotypes (Figure 6). Lower MGIDI values represent a shorter distance from the ideotype and, consequently, superior multi‐trait performance. Based on this criterion, genotype BR‐99, with the lowest MGIDI value (2.04), was identified as the most superior genotype, followed by RGC‐986 with an MGIDI value of 2.45, ranking second. Although genotypes such as S6566, S‐6581, and S‐6560 exhibited relatively low MGIDI values, only the first two genotypes (BR‐99 and RGC‐986) were classified as truly superior according to the selection intensity defined in the model. The remaining genotypes showed progressively higher MGIDI values, indicating an increasing distance from the desirable ideotype. In particular, genotypes RGC‐1031, RGC‐1066, and especially Pishen, with the highest MGIDI values (3.59, 4.26, and 5.36, respectively), demonstrated the weakest overall multi‐trait performance compared with the other genotypes.

**FIGURE 7:**
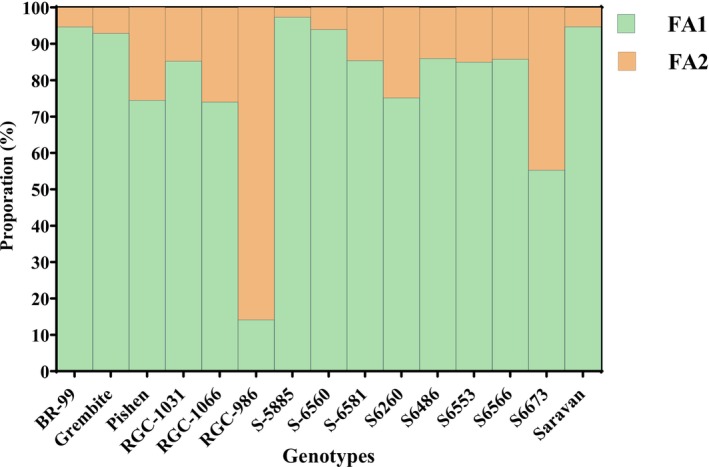
Percentage distribution of strengths and weaknesses of all genotypes across two factor analyses (FA) based on the MGIDI method.

The results of genotype contribution analysis to the extracted factors based on the MGIDI index revealed that the relative contributions of genotypes to the first two factors (FA1 and FA2) followed distinct patterns, reflecting substantial variation in the multi‐trait structure among guar genotypes (Figure 7). For most genotypes, the first factor (FA1) accounted for the dominant proportion of variation. Specifically, genotypes S‐5885, Saravan, BR‐99, S‐6560, and Grembite derived more than 92% of their total variation from FA1, indicating a strong alignment of these genotypes with the predominant traits defining the first factor. In contrast, genotype RGC‐986 exhibited a markedly different pattern, with the highest contribution attributed to the second factor (FA2), accounting for 85.84%, whereas the contribution of FA1 was estimated at only 14.16%. This pattern suggests a stronger dependence of RGC‐986 on a distinct set of traits loaded on FA2, which play a decisive role in the multi‐trait differentiation of this genotype. Some genotypes, such as S6673, showed a relatively balanced contribution pattern between the two factors, with FA1 and FA2 contributing 55.30% and 44.70%, respectively, indicating the simultaneous influence of both factors on the multi‐trait structure of this genotype. Additionally, genotypes Pishen, RGC‐1066, and S6260, which exhibited a higher contribution from FA2 compared with the other genotypes, demonstrated a greater divergence from the ideotype pattern defined by FA1.

### 3.5 Factor Analysis

The results of factor analysis indicated that the first two factors (FA1 and FA2) played a major role in explaining the variation of the evaluated traits and accounted for a substantial proportion of the total data variance (Table 2). The first factor (FA1), characterized by high and predominantly negative factor loadings, contributed most to the explanation of traits associated with nutritional quality, digestibility, and functional properties of gum. Specifically, seed gum percentage (SG), dry matter digestibility (DMD), fiber content (Fiber), protein content (Protein), total antioxidant capacity (TAC), viscosity (Viscosity), and carotenoids (Carotenoids) exhibited high loadings on FA1, with absolute values of 0.89, 0.96, 0.92, 0.88, 0.81, 0.72, and 0.77, respectively. In contrast, the second factor (FA2) was mainly influenced by tannin content (Tanin), which showed the highest positive loading on FA2 (0.86). This pattern indicates that variation in tannin content is largely independent of the other major traits and acts as a distinct axis in genotype discrimination. Viscosity also displayed a relatively high loading on FA2 (0.62), suggesting that this trait, in addition to its association with FA1, is partly influenced by the second factor. Examination of communality values revealed that most traits exhibited high communalities, with values exceeding 0.88 for traits such as viscosity, DMD, SG, fiber, and protein. This finding indicates that a large proportion of the variance in these traits is explained by the two extracted factors. In contrast, TAC showed a lower communality (0.66) and a higher uniqueness value (0.34), suggesting that part of its variation is governed by factors outside these two factors. Overall, the factor analysis results demonstrated that FA1, as the dominant factor, represents a set of key traits related to the nutritional and functional quality of guar, whereas FA2 primarily reflects independent variation associated with anti‐nutritional compounds such as tannins. This structural separation provides a robust basis for interpreting the MGIDI index results and for identifying genotypes that are closest to the ideotype.

**TABLE 2:** Table factor loadings, communalities, and unexplained variance for traits in factors analysis (FA1 to FA2) of results.

VAR	FA1	FA2	Communality	Uniquenesses
SG	−0.894	−0.304	0.891	0.109
Viscosity	−0.724	−0.624	0.914	0.086
DMD	−0.958	0.052	0.920	0.080
Fiber	−0.923	−0.173	0.882	0.118
TAC	−0.810	−0.062	0.660	0.340
Carotenoids	−0.765	0.397	0.743	0.257
Protein	−0.883	−0.316	0.879	0.121
Tannin	0.020	0.859	0.738	0.262

*Note:* See Table 1 for the definition of measured traits.

The results of predicted genetic gain for traits influencing the MGIDI index among the 15 guar genotypes indicated that selection of superior genotypes based on this index would lead to the simultaneous improvement of several key traits, particularly those loaded on the first factor (FA1) (Table 3). For seed gum percentage (SG), the initial population mean was 28.17, which increased to 31.09 after selection, resulting in a phenotypic gain of 2.92 units, equivalent to a 10.37% increase. Considering the high heritability of this trait, the realized genetic gain was estimated at 2.42 units, corresponding to an 8.58% increase, indicating a strong response of SG to MGIDI‐based selection. For viscosity, the highest predicted genetic gain was observed, with the mean increasing from 2644.93 to 3130.59, yielding a phenotypic gain of 485.66 units (18.36%). After accounting for high heritability, the realized genetic gain for this trait was estimated at 418.64 units, equivalent to a 15.83% increase, highlighting the major importance of viscosity in improving the industrial quality of guar gum. Analysis of dry matter digestibility (DMD) showed that selection increased the mean from 46.96 to 48.88, resulting in a phenotypic gain of 1.92 units (4.09%). The realized genetic gain for this trait was estimated at 1.24 units (2.64%), indicating a moderate but stable response of DMD to selection. Similarly, for fiber content (Fiber), the mean increased from 11.71 to 12.26, with a realized genetic gain of 0.42 units (3.55%), suggesting the potential for gradual improvement of this trait through multi‐trait selection. Regarding total antioxidant capacity (TAC), an increase in the mean from 44.90 to 45.95 was observed, corresponding to a realized genetic gain of 0.67 units (1.49%). In addition, despite a relatively narrow range of variation for carotenoids, a positive realized genetic gain of 2.27% was predicted, demonstrating the efficiency of the MGIDI index in improving bioactive traits even when phenotypic variation is limited. The results for protein content indicated a substantial response to selection, with the mean increasing from 28.07 to 30.68 and a realized genetic gain of 1.99 units (7.08%), emphasizing the importance of this trait in enhancing the nutritional value of guar. In contrast, tannin content, which was loaded on the second factor (FA2), showed a slight reduction in the mean from 2.43 to 2.40. The negative genetic gain of 1.06% for this trait indicates the effectiveness of the MGIDI index in reducing undesirable traits while simultaneously increasing desirable ones, which is considered one of the main advantages of ideotype‐based multi‐trait selection.

**TABLE 3:** Predicted genetic gain for traits influencing MGIDI index of the studied variables in guar ( *Cyamopsis tetragonoloba* ).

VAR	Factor	Xo	Xs	SD	SDperc	H2	SG	SGperc	Sense	Goal
SG	FA1	28.17	31.09	2.92	10.37	0.83	2.42	8.58	Increase	100
Viscosity	FA1	2644.93	3130.59	485.66	18.36	0.86	418.64	15.83	Increase	100
DMD	FA1	46.96	48.88	1.92	4.09	0.64	1.24	2.64	Increase	100
Fiber	FA1	11.71	12.26	0.55	4.68	0.76	0.42	3.55	Increase	100
TAC	FA1	44.90	45.95	1.05	2.35	0.64	0.67	1.49	Increase	100
Carotenoids	FA1	2.12	2.20	0.07	3.45	0.66	0.05	2.27	Increase	100
Protein	FA1	28.07	30.68	2.61	9.31	0.76	1.99	7.08	Increase	100
Tannin	FA2	2.43	2.40	−0.04	−1.51	0.70	−0.03	−1.06	Increase	0

### 3.6 Correlation

**FIGURE 8:**
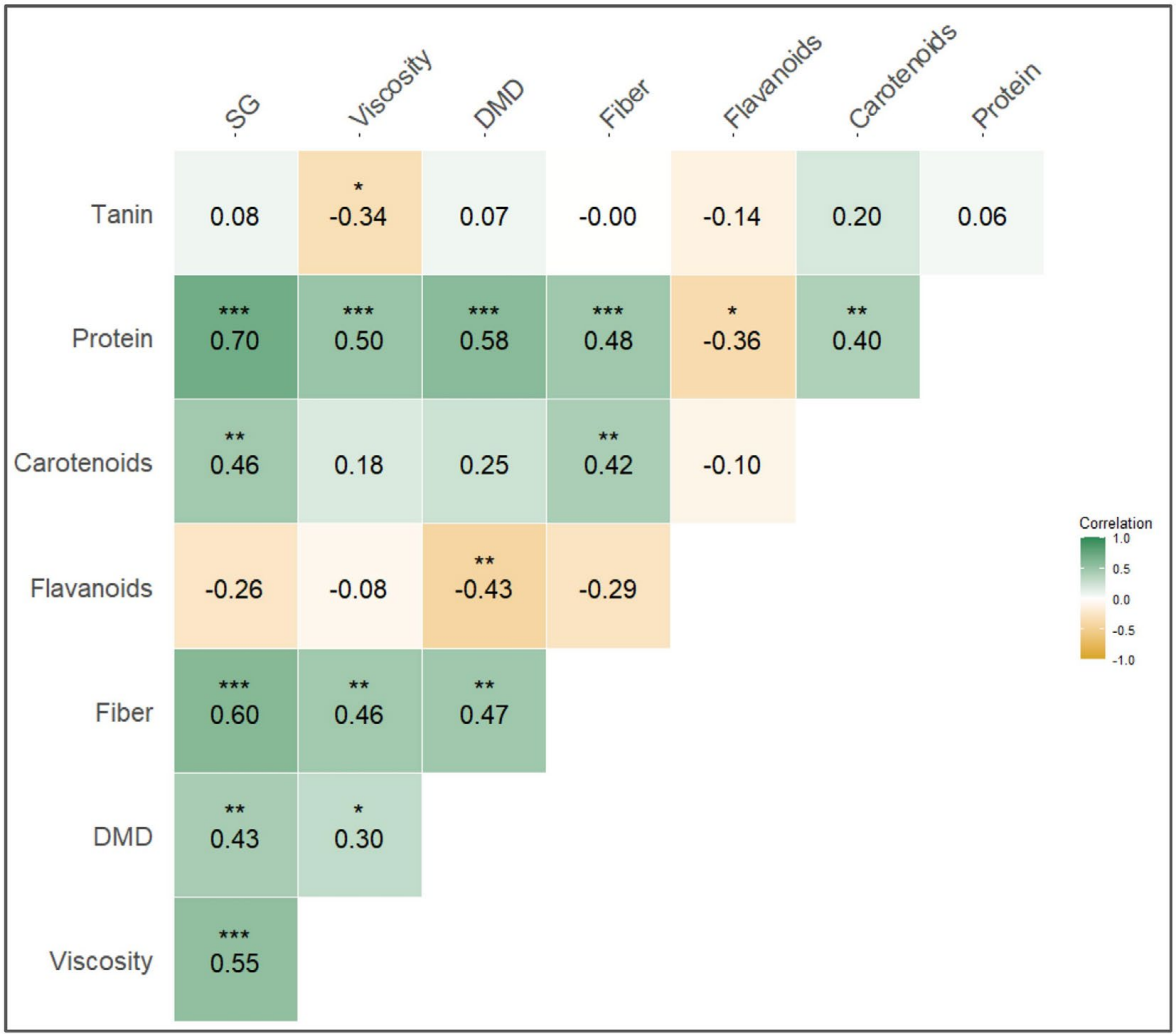
Pearson correlation coefficients of the studied variables in guar ( *Cyamopsis tetragonoloba* ) in 15 difference genotypes. The color scale, ranging from gold to green, represents correlations from highly positive to highly negative. The numbers in the figure indicate the Pearson correlation coefficients between the traits. Square stars denote the significance of the correlation coefficients (*p ≤ 0.05, **p ≤ 0.01, and ***p ≤ 0.001).

In this study, correlations among the evaluated traits were analyzed for those parameters that exhibited significant differences among the assessed genotypes (Figure 8). The results indicated that seed gum (SG) showed positive correlations with protein (0.70***), carotenoids (0.46**), fiber (0.60), dry matter digestibility (DMD; 0.43**), and viscosity (0.55***). In addition, viscosity exhibited a positive correlation with protein (0.50***) and fiber (0.46**), while showing a negative correlation with tannin content (−0.34*). Furthermore, DMD was negatively correlated with flavonoids (−0.43**) and positively correlated with protein (0.58) and fiber (0.47**). A positive correlation was also observed between fiber and protein (0.48**), as well as between fiber and carotenoids (0.42**). Moreover, protein content showed a negative correlation with flavonoids (−0.36*) and a positive correlation with carotenoids (0.40**).

## 4 Discussion

In the present study, Seed gum percentage (SG) among the 15 guar genotypes ranged from 25.7% to 32.8%, a variation that falls within the natural range of SG previously reported for guar germplasm. For instance, studies evaluating multiple guar genotypes have reported SG values ranging from 28.6% to 34.6%, indicating comparable levels of gum accumulation across diverse genetic materials. Similarly, Joshi et al. (2015) reported SG values varying from 26.12% to 31.46%, which closely aligns with the maximum SG observed in the present study (32.8%), suggesting a comparable genetic potential for gum production within guar germplasm (Joshi et al. 2015; Gresta et al. 2017). Previous investigations have also indicated that such variations in SG, even under uniform agronomic and environmental conditions, are predominantly attributable to genotypic differences rather than environmental effects, a finding that is consistent with the results of the current study (Gresta et al. 2017; Kotnala et al. 2024). From a mechanistic perspective, the observed genotypic variation in SG can be attributed to differences in the biochemical composition of the seed endosperm, as guar gum is primarily composed of galactomannan polysaccharides, and the mannose‐to‐galactose ratio within the endosperm has a direct influence on total gum content (Dzyubenko et al. 2023). Moreover, this biochemical structure is closely linked to the activity and expression of enzymes involved in the galactomannan biosynthetic pathway, such that genotypes exhibiting higher expression levels of these enzymes are capable of accumulating greater amounts of gum in the endosperm (Tahmouzi et al. 2023).

The genotypic differences in SG observed in this study are of considerable importance from a plant breeding perspective, as they demonstrate that gum yield is a genetically selectable trait, and genotypes with higher SG can be effectively utilized as parental lines to improve gum yield in guar breeding programs (Gresta et al. 2017). From an applied and industrial standpoint, as well as in terms of nutritional value, higher SG is directly associated with improved processing efficiency and enhanced quality of the final guar gum product, since higher gum content contributes to superior thickening and stabilizing properties in food formulations and various industrial applications (Ashraf et al. 2002; Li et al. 2023).

The observed positive correlations among seed gum (SG), protein, fiber, viscosity, and dry matter digestibility (DMD) reflect an integrated biological framework governing seed composition in guar. The strong association between SG and viscosity is expected, as both traits are directly influenced by the quantity and structural characteristics of galactomannan stored in the endosperm. Genotypes with higher SG likely possess enhanced biosynthetic activity of galactomannan, leading not only to increased gum accumulation but also to improved rheological properties due to greater molecular weight and polymer interactions. Moreover, the positive correlation between protein and DMD suggests that seeds with higher storage protein content may exhibit improved enzymatic degradability, enhancing nutritional quality. The association of fiber with both SG and DMD further indicates that the dominant fiber fraction in guar, primarily soluble galactomannan, contributes positively to digestibility, unlike insoluble fibers that typically reduce it. In contrast, the negative correlations between flavonoids and protein or DMD suggest a metabolic shift toward secondary metabolite production at the expense of primary storage compounds, reflecting competition for carbon resources during seed development (Joshi et al. 2015). In that study, viscosity values varied from 2104 to 6240 mPa·s, supporting the notion that viscosity is a highly variable trait influenced by genetic background. This molecular architecture promotes extensive intermolecular interactions and increases resistance to flow in aqueous solutions (Malani et al. 2024). Previous studies have shown that the viscosity of guar gum solutions at a concentration of 1% can reach values as high as 10,000 cP, depending largely on molecular weight, the mannose‐to‐galactose ratio, and the degree of polymer branching (Mudgil et al. 2014; Malani et al. 2024). In this context, the viscosity values observed in the present study (2353–3466 cP) fall within the reported range for 1% guar gum solutions, although the evaluated genotypes may differ in polymer molecular weight, which likely explains the observed variation.

The presence of significant differences in viscosity among the 15 studied genotypes is of considerable importance, as rheological traits such as viscosity are closely associated with the genetic composition of the seed endosperm. Therefore, selecting genotypes with higher viscosity can facilitate the development of improved breeding lines with superior gum quality (Joshi et al. 2015). From an applied and industrial perspective, viscosity is not merely a physiological trait but a key determinant of guar gum functionality in both food and non‐food processing. Gums exhibiting higher viscosity at lower concentrations are particularly desirable for improving texture, stabilizing emulsions, and controlling consistency in baked products and beverages, which explains the broad industrial acceptance of high‐viscosity guar gum (Sharma et al. 2018).

Differences in DMD among genotypes may reflect variations in the structural composition of endosperm carbohydrates and proteins, as both the proportion and type of fibrous components (e.g., cellulose and hemicellulose) and protein content directly influence enzymatic accessibility during digestion (Antil and Chhikara 2019). Other studies have similarly demonstrated that dry matter digestibility is strongly dependent on the chemical composition of guar seeds or guar‐based concentrates. In particular, higher levels of insoluble fiber tend to reduce DM digestibility, whereas increased protein content and readily accessible components enhance DMD (Dodi et al. 2011; Sharma et al. 2018; Tahmouzi et al. 2023; Zheng et al. 2023). Overall, the observed differences in DMD among the evaluated guar genotypes indicate that the internal chemical composition of guar seeds, especially the balance between digestible and indigestible fractions, plays a critical role in determining DMD and can be effectively exploited in breeding programs aimed at improving the nutritional value of guar (Antil and Chhikara 2019).

The evaluation of total oil content (TO) among guar ( *Cyamopsis tetragonoloba* ) genotypes showed that this trait varied from 2.23% in the Pishen genotype to 3.27% in RGC‐986. In line with these findings, Gresta et al. (2017) reported that lipids, expressed on a dry matter basis, averaged 28.3 g kg^−1^ DM, with significantly higher values observed in genotypes originating from South Africa and India. In guar seeds, the majority of oil is stored as essential unsaturated fatty acids, and the distribution of fatty acids, particularly linoleic and oleic acids, can be strongly influenced by genetic factors, ultimately affecting total oil content (Chiofalo et al. 2018). This observation is consistent with reports of significant variation in both lipid content and fatty acid composition among seeds of different legume species, including guar (Gresta et al. 2017). Although oil represents a relatively small fraction of guar seed composition, it has been reported to contain nutritionally valuable polyunsaturated fatty acids beneficial for human and animal nutrition; therefore, increasing TO may enhance the nutritional value of guar‐based food and feed products.

The analysis of tannin content among the 15 guar genotypes revealed values ranging from 2.06% in RGC‐986 to 2.70% in BR‐99, which falls within the range reported in previous guar studies, although exact tannin concentrations may vary depending on sample type and analytical method. A comprehensive quantitative profiling of guar seed extracts reported tannin levels below the lower limit of quantification (i.e., < 1.5 mg catechin equivalents g^−1^), indicating generally low to moderate tannin content in guar seeds (Chiofalo et al. 2018). More detailed investigations have shown that, when several guar varieties were evaluated under Mediterranean conditions, tannins in guar seed kernels were often below the UV detection threshold compared with other phenolic compounds, suggesting that tannins may represent a relatively minor component of the overall phenolic profile of guar (Sharma, Joshi, et al. 2017; Sharma, Kaur, and Kaur 2017).

The assessment of protein content among the 15 guar genotypes demonstrated values ranging from 25.33% in Pishen to 33.33% in RGC‐986, which is consistent with previously reported ranges for guar seed protein in nutritional studies, where values of approximately 24%–34% have been documented (Vishwakarma et al. 2012). Eldirany et al. (2018) reported even higher protein contents (37%–42.8%) based on proximate analysis of seeds from four different guar genotypes. In addition, Gresta et al. (2017) reported an average protein content of 283 g kg^−1^ DM across guar genotypes, based on a dry matter content of 896 g kg^−1^.

Based on the results of the present study, crude fiber content among the 15 guar ( *Cyamopsis tetragonoloba* ) genotypes ranged from 10.57% in the Pishen genotype to 12.95% in RGC‐986, indicating substantial genotypic variation in seed fiber composition. Previous studies have likewise reported that guar seeds contain considerable amounts of crude fiber; for instance, evaluations of multiple guar genotypes have shown crude fiber contents ranging approximately from 10% to 12%, which closely aligns with the variation observed in the present investigation and confirms that guar inherently possesses a relatively high fiber content (Gresta et al. 2017).

In a study conducted on several guar accessions, TDF and SDF accounted for approximately 52%–58% and 26%–32% of seed dry weight, respectively, highlighting the dominant contribution of both soluble and insoluble fiber fractions to guar seed structure (Kays et al. 2006). Although earlier reports, such as those by Sharma, Joshi, et al. (2017); Sharma, Kaur, and Kaur (2017) documented slightly lower crude fiber values (approximately 8.44%–9.81%), the higher fiber content observed in the present study may be attributed to genotypic diversity, environmental conditions, or differences in analytical methodologies. The analysis of TPC among the 15 guar genotypes revealed values ranging from 0.87 in genotype S6486 to 1.26 in genotype S‐6560, reflecting notable genotypic variability in phenolic compound accumulation. Similarly, Gresta et al. (2017) reported total polyphenol contents ranging from 3.61 to 6.63 mg gallic acid equivalents (GAE) g^−1^, which were higher than those observed in the present study. Comparable trends have also been reported by Joshi et al. (2023), supporting the existence of significant variability in phenolic composition among guar genotypes.

Carotenoids are natural pigmented compounds in plants that act as antioxidants, helping protect cells against environmental stresses and enhancing photosynthesis (Habibi et al. 2025). Analysis of carotenoid content revealed values ranging from 1.50 in Pishen to 2.47 in RGC‐986, reflecting genotypic differences in carotenoid accumulation within guar seeds. Similar variability has been reported in other legumes, suggesting that carotenoid content is strongly influenced by genetic background (Padhi et al. 2017). Such variation may result from differences in carotenoid biosynthetic pathways and expression of genes involved in lutein and β‐carotene synthesis, as demonstrated in genetic studies of chickpea and other legumes (Tahmouzi et al. 2023; Padhi et al. 2017). The observed diversity in carotenoid content among guar genotypes indicates substantial genetic potential for enhancing these bioactive compounds through targeted breeding strategies aimed at improving nutritional quality.

The multi‐trait genotype–ideotype distance index (MGIDI) analysis identified genotypes BR‐99 and RGC‐986 as superior, exhibiting the lowest MGIDI values (2.036 and 2.452, respectively), and thus showing the closest multi‐trait performance to the desirable ideotype. This outcome reflects the core principle of the MGIDI approach, which prioritizes genotypes with balanced performance across multiple traits (Olivoto and Nardino 2021). The MGIDI index is particularly valuable because it integrates multivariate analysis and dimensionality reduction (e.g., factor analysis) to estimate the distance of each genotype from an ideal combination of traits, enabling more efficient selection than single‐trait indices.

From a breeding perspective, the clustering of SG, viscosity, protein, fiber, and DMD within the same factor (FA1) provides strong evidence that these traits are genetically and physiologically interconnected and can be improved simultaneously through selection. This is particularly important because it indicates the absence of strong antagonistic relationships among key economic traits. The MGIDI index further supports this conclusion, as the superior genotypes (BR‐99 and RGC‐986) exhibited balanced performance across both nutritional and functional traits. Therefore, these genotypes represent promising parental material for breeding programs aimed at developing high‐yielding guar cultivars with enhanced gum quality and nutritional value. Additionally, the separation of tannin into an independent factor (FA2) suggests that it is possible to reduce anti‐nutritional compounds without negatively affecting the main desirable traits, which is a critical objective in the development of food‐ and feed‐grade guar varieties (Habibie et al. 2019; Joshi et al. 2023).

The superior genotypes identified in this study offer distinct opportunities for practical applications. Genotype BR‐99, characterized by high protein, fiber, and antioxidant‐related traits, is particularly suitable for food and feed applications, including functional foods and protein‐rich formulations. In contrast, RGC‐986, with its high SG and viscosity, is more appropriate for industrial uses where thickening and stabilizing properties are critical, such as in food processing, pharmaceuticals, and oil industries. The positive association among several key traits also suggests the feasibility of developing dual‐purpose guar cultivars that combine both high nutritional quality and desirable industrial properties, thereby expanding the utilization potential of this crop.

## 5 Conclusion

This study demonstrated substantial genetic variability among 15 guar ( *Cyamopsis tetragonoloba* L.) genotypes in key nutritional, biochemical, and functional traits, highlighting the decisive role of genetic background in determining seed quality. Significant differences were observed in protein, fiber, antioxidant‐related compounds, viscosity, and digestibility, confirming guar as a protein‐rich legume with strong potential for food, feed, and industrial applications. Multivariate analyses, including PCA, effectively discriminated genotypes and associated quality traits, while MGIDI identified BR‐99 and RGC‐986 as superior genotypes exhibiting consistently desirable nutritional and functional profiles. Overall, the observed diversity provides valuable opportunities for targeted breeding programs aimed at enhancing nutritional quality, minimizing antinutritional factors, and improving industrial performance, thereby supporting the broader utilization of guar in food systems and bioproduct development.

## Author Contributions

**Warqaa Muhammed ShariffAl‐Sheikh:** conceptualization, software, formal analysis, validation. **Ahmed Fahem Jabbar:** investigation, validation, visualization, project administration, methodology. **Heidar Meftahizade:** conceptualization, writing – review and editing, project administration, supervision, data curation, methodology. **Hussam F. Najeeb Alawadi:** investigation, visualization, funding acquisition, project administration.

## Funding

The authors have nothing to report.

## Ethics Statement

The authors have nothing to report.

## Consent

The authors have nothing to report.

## Conflicts of Interest

The authors declare no conflicts of interest.

## Data Availability

The data that support the findings of this study are available from the corresponding author upon reasonable request.
